# Oral Spore-Based Probiotic Supplementation Alters Post-Prandial Expression of mRNA Associated with Gastrointestinal Health

**DOI:** 10.3390/biomedicines12102386

**Published:** 2024-10-18

**Authors:** Brian K. McFarlin, Sarah E. Deemer, Elizabeth A. Bridgeman

**Affiliations:** 1 Applied Physiology Laboratory, University of North Texas, Denton, TX 76205, USA; sarah.deemer@unt.edu (S.E.D.); elizabeth.bridgeman@unt.edu (E.A.B.); 2 Department of Biological Sciences, University of North Texas, Denton, TX 76205, USA

**Keywords:** metabolic endotoxemia, chronic disease, high-fat meal, leaky gut syndrome

## Abstract

**Background/Objectives**: Unregulated post-prandial dietary endotoxemia may accumulate over time and underlie the development of chronic disease (e.g., leaky gut, inflammatory bowel disease, etc.), for which oral probiotic supplementation may be a prophylactic. The purpose of this study was to determine if 45 d of oral spore-based probiotic supplementation altered gastrointestinal-associated mRNA expression following a high-fat meal. **Methods**: A subset of apparently healthy individuals from a larger study who had dietary endotoxemia at baseline completed 45 d of supplementation with either a placebo (rice flour; *n* = 10) or spore-based probiotic (Megasporebiotic™; Novonesis, Kongens Lyngby, Denmark; *Bacillus indicus* (HU36™), *Bacillus subtilis* (HU58™), *Bacillus coagulans* (SC208™), and *Bacillus licheniformis* (SL-307), and *Bacillus clausii* (SC109™); *n* = 10). Venous blood was collected in Paxgene RNA tubes prior to (PRE), 3 h, and 5 h after consumption of a high-fat meal (85% of the daily fat RDA and 65% of the daily calorie needs). Total RNA was analyzed for 579 mRNAs of interest (Nanostring nCounter Sprint; Seattle, WA, USA). After normalization to housekeeping controls and calculation of differential expression relative to PRE and controlled for FDR, 15 mRNAs were determined to be significantly changed at either 3 h and/or 5 h post-prandial in the probiotic group but not in the placebo group. **Results**: Significant mRNA expressions were associated with gastrointestinal tract barrier function (four mRNAs: BATF3, CCR6, CXCR6, and PDCD2), gastrointestinal immunity (four mRNAs: CLEC5A, IL7, CARD9, and FCER1G), or future IBD risk (seven mRNAs: PD-L1, CSF1R, FAS, BID, FADD, GATA3, and KIR3DL). **Conclusions**: Collectively, the present findings may support the notion that post-prandial immune response to eating is enhanced following 45 d of probiotic supplementation.

## 1. Introduction

A significant proportion of the young adult population experiences transient dietary endotoxemia during the first five post-prandial hours [1,2]. Dietary endotoxemia is characterized by transient disruption of gastrointestinal barrier function following consumption of a high-fat/high-calorie meal [2]. In early adult life, post-prandial dietary endotoxemia and the subsequent immune response are short-term, transient in nature. Unfortunately, if left unmanaged, transient dietary endotoxemia becomes chronic, leading to a variety of chronic disease states (e.g., metabolic syndrome, cardiovascular disease, inflammatory bowel disease (IBD), colorectal cancer, etc.) [3,4,5,6]. The pharmaceutical industry has devoted significant effort and resources toward the development of treatments for fully progressed IBD; however, more research is needed to understand how prophylactic lifestyle, physical activity, and nutritional interventions reduce the incidence of dietary endotoxemia and potentially future IBD risk [7,8,9]. It is also important to note that patients with IBD tend to have very low physical activity levels, further exacerbating endotoxemia [10,11]. Prophylactic treatments with the potential to rapidly enhance and then reduce the immune system’s response to transient dietary endotoxemia are needed.

Our laboratory and others have used oral probiotic supplementation to reduce dietary endotoxemia [1,2,7,12]. Specifically, we have previously reported that 30 d of spore-based probiotic supplementation resulted in a 42% reduction in the incidence of dietary endotoxemia in participants who had dietary endotoxemia at baseline [2]. We also previously reported that overweight adults who supplemented for 90 d with a spore-based probiotic had reduced mRNA expression associated with adipose tissue inflammation, systemic inflammation, and chronic disease risk [1]. Our approach and previous findings regarding probiotics are consistent with what others have reported [1,2]. Also, probiotic fungi have recently been purported to modulate chronic inflammation in IBD models [13]. Despite the promising findings from our lab and others, knowledge gaps related to probiotics as a possible prophylactic persist [7]. The present study sought to partially address these gaps using a panel of mRNA expression biomarkers. An advantage of mRNA biomarkers is that they tend to be more sensitive to smaller changes than protein biomarkers, allowing for a smaller sample size to detect significant changes [1,14,15,16,17,18]. The purpose of the present study was to determine how 45 d of spore-based probiotic supplementation altered post-prandial expression of mRNA associated with gastrointestinal tract health following consumption of a high-fat/calorie meal.

## 2. Materials and Methods

### 2.1. Institutional Review Board Approval

The present study was conducted according to the latest Declaration of Helsinki, and all protocols were approved by the University of North Texas Institutional Review Board. All participants provided oral and written consent to participate in the present study. This study included a subset from a larger study being conducted in the laboratory to evaluate other impacts of probiotic supplementation beyond gastrointestinal health and IBD.

### 2.2. Participants, Sample Size, and Inclusion/Exclusion Criteria

The present analysis was designed based on our previous publications involving similar probiotic blends used in the present study [1,2]. We also used other studies we have conducted using Nanostring mRNA analysis to estimate a priori sample size using compared comparisons in G-power v. 3.1.9.7 [1,2,15,16,17,18]. Using our previous studies, we determined that we needed a minimum of 9 participants with dietary endotoxemia in each condition to achieve a statistical power of 0.80. A statistical power of 0.80 reflected a differential expression level of at least 0.25 between placebo and probiotic at either 3 h or 5 h post-prandial. As the samples for the present study were derived from a large study that did not focus on individuals with dietary endotoxemia, we analyzed the participant baseline from the larger study (prior to supplementation) for dietary endotoxemia using our previous criteria [2]. Since we only examined participants with dietary endotoxemia, it is plausible that the application of our findings is limited to individuals with this condition. This identified 20 individuals who had been randomized to the placebo (*n* = 10) or probiotic (*n* = 10) group and had completed all study visits and requirements. The 20 individuals were of normal body weight (BMI = 26.9 ± 1.5 kg/m^2^), young (20.9 ± 0.6 y), had no incidence of diagnosed disease, and were taking no medications on a regular basis (values are mean ± SEM). This number of participants exceeded the minimum per group we calculated to yield at least 0.80 statistical power. 

In order to be enrolled in the study, participants had to be between the ages of 18 and 35 y; not participating in another research study; not be a current or previous (past 6 months) tobacco user; not have a diagnosed metabolic or inflammatory disease; not currently be taking a probiotic supplement (in the past 6 months); not be regularly consuming yogurt with probiotics; not be allergic to probiotics, rice, and/or cheese; not be taking over the counter medications on a daily basis; not have taken antibiotics in the past 6 months; not be trying to lose or gain weight; not be pregnant or trying to become pregnant; not be breast feeding/lactating; and not be anemic.

### 2.3. Supplementation Conditions

The spore-based probiotic used in the present study was commercially manufactured (Megasporebiotic™; Novonesis, Kongens Lyngby, Denmark) and included 4 billion spores from the following Gram-positive, spore-forming strains: *Bacillus indicus* (HU36™), *Bacillus subtilis* (HU58™), *Bacillus coagulans* (SC208™), *Bacillus licheniformis* (SL-307), and *Bacillus clausii* (SC109™). The probiotic was stable and stored at room temperature in the participant’s home, where they were instructed to consume the supplement at approximately the same time each day. Participants were provided their supplement (4 capsules per day) in a daily dose blister pack format for a total of 45 d. Rice flour was used in both the probiotic and placebo conditions as a loading agent. Based on subject reporting, the efficacy of intake was >90% for the study period. All group assignments were completed using double-blind procedures.

### 2.4. Experimental Meal Challenge

We used a similar methodology as described previously [2]. Briefly, following the 45 d supplementation period, participants reported to the laboratory between 06:00 and 10:00 h following an overnight fast (>8 h) and abstention from exercise (>24 h). Following collection of a pre-meal blood sample, subjects were provided a high-fat meal (85% of the daily fat RDA and 65% of the daily calorie needs) that was adjusted based on the participant’s measured daily caloric needs (calculated by the Harris–Benedict equation). A frozen, thin-crust cheese pizza was used as the high-fat meal source and had a nutritional content like our previous study [2].

### 2.5. Blood Collection and RNA Extraction

Venous blood samples were collected prior to the high-fat meal (PRE), 3 h, and 3 h post-prandial from a peripheral arm vein into a Paxgene mRNA tube (PreAnalytiX, Hombrechtikon, Switzerland). Paxgene blood samples were mixed by inversion, frozen at −20 °C for 24 h, and subsequently transferred to a −80 °C freezer until RNA extraction. Sample preparation and RNA extraction were performed according to previously described procedures [1,15,16,17,18,19]. Total RNA was extracted from PAXgene blood samples using a commercially available isolation kit (PAXgene^®^ Blood miRNA kit; PreAnalytiX, Hombrechtikon, Switzerland) with an automated isolation system (QIAcube; Qiagen, Hilden, Germany). The isolated RNA concentration was standardized to 20 ng/µL in nuclease-free water.

### 2.6. Nanostring mRNA Expression Analysis

Total RNA was analyzed for the expression of 579 mRNAs using a commercially available panel (Human Immunology v2 Panel; NanoString, Seattle, WA, USA) following the manufacturer’s instructions. The Nanostring CodeSets (reagents) used in this study included a collection of target mRNA, positive control mRNA, negative control mRNA, and housekeeping control mRNA. Raw image counts were obtained using a Sprint Profiler (nCounter; NanoString, Seattle, WA, USA). Raw files were processed further for normalization and significance using HyperScale ROSALIND^®^ (https://rosalind.onramp.bio/, accessed on 15 October 2024) (ROSALIND, Inc., San Diego, CA, USA).

### 2.7. mRNA Expression Normalization and Identification

**Figure 1:**
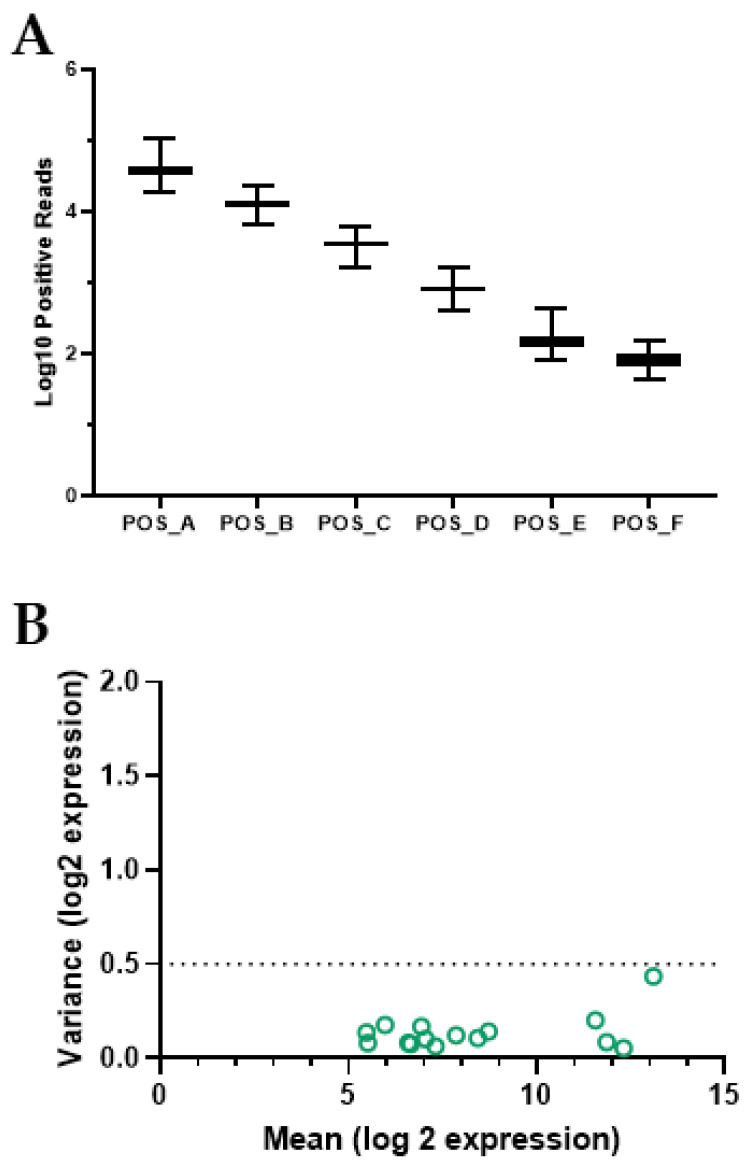
mRNA quality control. Quality control data are presented for determination of mRNA expression using a Nanostring nCounter multiplex system. Panel (**A**) presents the Log10 counts for the 6 positive controls spiked into each sample. The expression of the controls was within manufacturer’s parameters. Panel (**B**) presents the variance in expression for 40 housekeeping mRNAs spiked into each sample. 32 housekeeper mRNAs were used for normalization (green circles)-based QC analysis completed using the geNorm algorithm.

Differential mRNA expressions were calculated for each supplement condition (probiotic and placebo) using PRE as the comparison point (i.e., setting PRE as a zero change). We then identified mRNAs that were significantly expressed in the probiotic group and not the placebo group. Specifically, mRNA was expressed as the log2 fold change from the corresponding PRE value to normalize the response to a 0 center and indicate the direction of expression (i.e., up- or down-regulated). Significance was set at *p* < 0.05 for all comparisons. As part of the analysis, routine quality control testing was conducted to ensure that the assay results were consistent with the manufacturer’s recommended ranges (Figure 1). A small number of samples (8 of 60) were excluded from further expression analysis because the expression of 70% of their respective housekeeping controls was below 50 counts. The significant outcomes are presented as volcano plots and standard bar graphs.

### 2.8. Data Processing and False Discovery Control

The data were analyzed with the nCounter^®^ Advanced Analysis protocol (NanoString) to generate sample quality control analysis (Figure 1). The NanoString criteria were used for normalization, fold changes, and *p*-value calculations. Advanced Analysis divided counts for a sample by the geometric mean of the normalization probes from the same sample. Nanostring does not recommend the universal application of multiple-comparison corrections due to their inherent experimental control and the risk of false negative inflation. false discovery rate (FDR) is a control for a Type I error that can occur when multiple comparisons are completed. Thus, we used a robust approach to limit FDR due to multiple comparisons while not inflating the false negative rate. In the present study, inflation of false negative rate would be equally detrimental to false positives. To address and remove false positives prior to manuscript generation, we examined the mRNAs identified as significant and systematically removed mRNAs that were likely to be false positives based on the following criteria: 1) the fold change in expression was less than 0.5 or greater than −0.5, 2) the fold change in expression was similar in the placebo and probiotic groups (indicating no treatment effect), and/or 3) the significant mRNAs had no support in the published literature linking them to probiotics, inflammation, chronic disease, etc. Initially 37 mRNAs were identified as significant; however, 22 mRNAs were removed using our false positive control procedures. This resulted in the identification of 15 mRNAs that were significantly changed in the probiotic group but not the placebo group post-prandial.

## 3. Results

### 3.1. Overview of Findings

Participants who had dietary endotoxemia at baseline were included in this analysis after having been randomized to complete either 45 d of a probiotic or placebo intervention. After analysis and control for false positive and false negative discovery, we reviewed the existing literature and classified the significant mRNAs to the following responses: (1) gastrointestinal tract barrier function, (2) gastrointestinal tract immunity, or (3) future risk of IBD.

### 3.2. Gastrointestinal Tract Barrier Function

**Figure 2:**
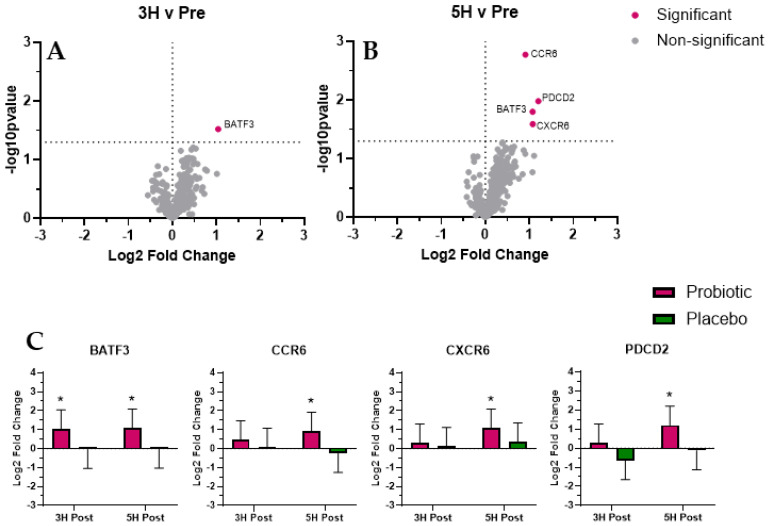
Gastrointestinal tract barrier function. Volcano plots present differential expression of probiotic mRNA expression pattern at 3H (**A**) and 5H (**B**). Additional bar plots (**C**) represent mRNA expression response for mRNA that reached significance for probiotic. Total RNA and subsequent mRNA expression analysis was completed using PAXgene whole blood was used as the RNA source. We found four mRNA whose expression reached significance (adjusted *p* < 0.05) and were associated with GI barrier function: BATF3, CCR6, CXCR6, and PDCD2. The values in the volcano plot and bar graphs are presented as Log2 fold changes, and the significance is shown as the –Log10 *p*-value. * Indicates significant difference (adjusted *p* < 0.05).

We identified four mRNAs whose expression was significantly (*p* < 0.05; Figure 2) altered with probiotic use and were associated with gastrointestinal tract barrier function: Basic leucine zipper transcription factor, ATF-like 3 (BATF3; increased expression at 3 h and 3 h); Chemokine (C-C motif) receptor 6 (CCR6; increased at 5 h); Chemokine (C-X-C motif) receptor 6 (CXCR6; increased at 5 h); and Programmed cell death 2 (PDCD2; increased at 5 h).

### 3.3. Gastrointestinal Tract Immunity

**Figure 3:**
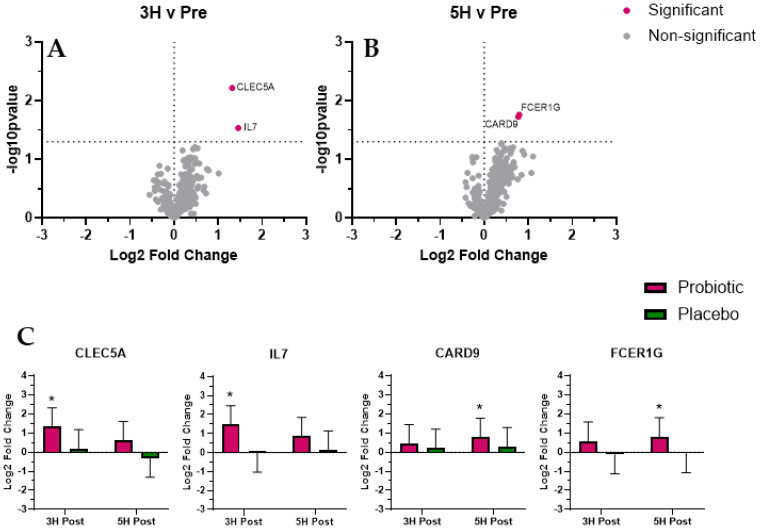
Gastrointestinal tract immunity. Volcano plots present differential expression of probiotic mRNA expression pattern at 3H (**A**) and 5H (**B**). Additional bar plots (**C**) represent mRNA expression response for mRNA that reached significance for probiotic. Total RNA and subsequent mRNA expression analysis was completed using PAXgene whole blood was used as the RNA source. We found four mRNA whose expression reached significance (adjusted *p* < 0.05) and were associated with GI immunity: CLEC5A, IL7, CARD9, and FCER1G. The values in the volcano plot and bar graphs are presented as Log2 fold changes, and the significance is shown as the –Log10 *p*-value. * Indicates significant difference (adjusted *p* < 0.05).

We identified four mRNAs whose expression was significantly (*p* < 0.05; Figure 3) altered with probiotic use and were associated with gastrointestinal tract immunity: C-type lectin domain family 5, member A (CLEC5A; increased at 3 h); Interleukin 7 (IL7; increased at 3 h); Caspase recruitment domain family, member 9 (CARD9; increased at 5 h); and Fc fragment of IgE, high affinity I, receptor for gamma polypeptide (FCER1G; increased at 5 h).

### 3.4. Future Risk of IBD

**Figure 4:**
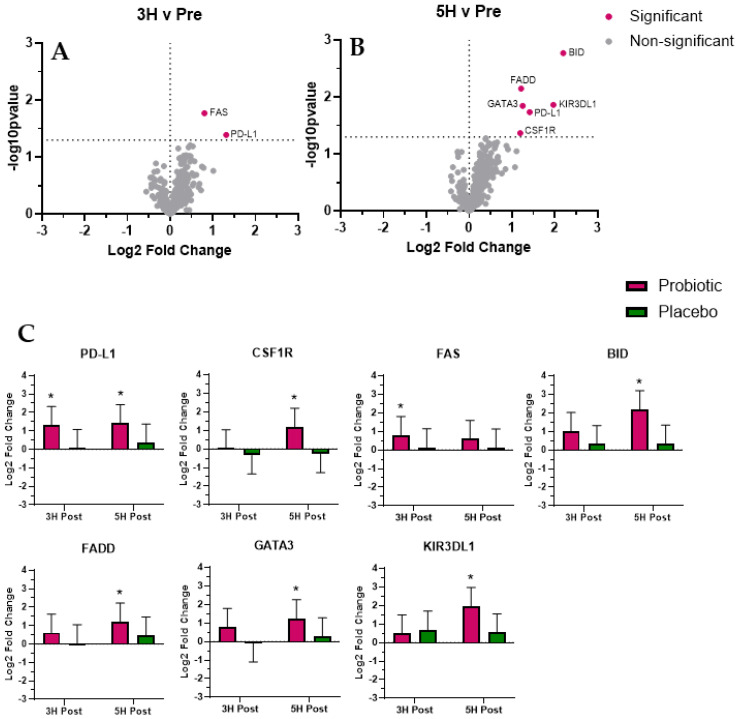
Future risk of IBD. Volcano plots present differential expression of probiotic mRNA expression pattern at 3H (**A**) and 5H (**B**). Additional bar plots (**C**) represent mRNA expression response for mRNA that reached significance for probiotic. Total RNA and subsequent mRNA expression analysis was completed using PAXgene whole blood was used as the RNA source. We found seven mRNA whose expression reached significance (adjusted *p* < 0.05) and were associated with Future risk of IBD: PD-L1, CSF1R, FAS, BID, FADD, GATA3, and KIR3DL1. The values in the volcano plot and bar graphs are presented as Log2 fold changes, and the significance is shown as the –Log10 *p*-value. * Indicates significant difference (adjusted *p* < 0.05).

We identified seven mRNAs whose expression was significantly (*p* < 0.05; Figure 4) altered with probiotic use and were associated with future risk of IBD: Programmed Death Ligand 1 (PD-L1; increased at 3 h and 5 h); Colony stimulating factor 1 receptor (CSF1R; increased at 5 h); TNF receptor superfamily, member 6 (FAS; increased at 3 h); BH3 interacting domain death agonist (BID; increased at 5 h); Fas (TNFRSF6)-associated via death domain (FADD; increased at 5 h); GATA binding protein 3 (GATA3; increased at 5 h); and killer cell immunoglobulin-like receptor, three domains, long cytoplasmic tail, 1 (KIR3DL1; increased at 5 h).

## 4. Discussion

The primary objective of the present study was to identify mRNAs whose expression was altered post-prandial following consumption of a high-fat/calorie meal after 45 d of oral spore-based probiotic supplementation. We focused on mRNA targets whose expression were associated with gastrointestinal functions and/or may be predictive of future risk of IBD. To that end, we identified 15 mRNAs whose expression was uniquely changed at either 3 h or 5 h post-prandial following 45 d of spore-based probiotic supplementation. Based on the existing literature, these mRNAs were associated with gastrointestinal tract barrier function (four mRNAs: BATF3, CCR6, CXCR6, and PDCD2), gastrointestinal immunity (four mRNAs: CLEC5A, IL7, CARD9, and FCER1G), and future risk of IBD (eight mRNAs: PD-L1, CSF1R, FAS, BID, FADD, GATA3, and KIR3DL). In accomplishing this objective, we speculate that the present findings may reflect an enhanced ability of the immune system to respond to dietary endotoxemia following probiotic supplementation. It is important to acknowledge that all our participants were apparently healthy, young individuals who had not been diagnosed with any disease but were positive for dietary endotoxemia at baseline. To our knowledge, we are the first to report that probiotic supplementation transiently altered post-prandial mRNA expression of targets associated with various aspects of gastrointestinal tract health. Future research may seek to explore longer courses of probiotic supplementation to determine the stability of the observed changes over time.

### 4.1. Gastrointestinal Tract Barrier Function

We identified four mRNAs (BATF3, CCR6, CXCR6, and PDCD2) whose expression was significantly altered during the post-prandial period following probiotic supplementation and associated with gastrointestinal tract barrier function. Mice with a knockout of Basic leucine zipper transcription factor, ATF-like 3 (BATF3) have disrupted gut barrier function, inefficient response to oral immunization, and increased susceptibility to metabolic syndrome [20,21]. Also, increased expression of Programmed cell death 2 (PDCD2) maintains gut barrier cells and limits the formation of gastrointestinal stromal tumors [22]. The severity of gut barrier disruption was exacerbated when mice were placed on a chronic, high-fat diet [22]. In response to antigens, an increased BATF3 expression prevents T-cell exhaustion, thus improving immunity [23]. In the present study, we observed increased BATF3 (3 h and 5 h) and PDCD2 (5 h only) with probiotic use, which collectively may represent a beneficial effect for transient gut barrier function. Given their respective physiological functions, it is plausible that the observed changes in BATF3 and PDCD2 may also translate to improved oral immunization when given in combination with probiotics.

Chemokine (C-C motif) receptor 6 (CCR6) regulates transmigration of Th17 and Treg cells to the skin and mucosal barriers [24,25], which may play a role in the development of IBD. Also, patients with active IBD tend to have suppressed expression of both CCR6 and Chemokine (C-X-C motif) receptor 6 (CXCR6) [25], which underlies gut barrier disfunction. In the context of the present study, we examined CCR6 and CXCR6 expression to evaluate the efficiency of post-prandial gut barrier immune function against dietary endotoxemia. We specifically found that probiotic use was associated with a significant increase in both CCR6 and CXCR6 expression 5 h post-prandial. More research is needed to fully understand what transient changes in CCR6 and CXCR6 indicate with respect to chronic disease onset. When combining the observed probiotic-associated changes in BATF3, CCR6, CXCR6, and PDCD2 expression, it appears that 45 d of probiotic supplementation may improve post-prandial gut barrier function, which reflects a beneficial adaptation. More research is needed to evaluate if the short-term changes we observed in the present study are stable over longer periods of time and reflective of reduced IBD risk.

### 4.2. Gastrointestinal Tract Immunity

We identified four mRNAs (CLEC5A, IL7, CARD9, and FCER1G)) whose expression was significantly altered during the post-prandial period following probiotic supplementation and associated with gastrointestinal immunity. Strength of the gastrointestinal immune response involves mucosal barrier function as well as the capacity and localization of effector leukocytes. C-type lectin domain family 5, member A (CLEC5A) is a pattern recognition receptor that works in tandem with TLR2 on the macrophage to detect and bind a variety of bacteria [26,27]. Given this role, it is important in host defense against infection and limiting dietary endotoxemia. In the present study, we found that probiotic supplementation increased CLEC5A expression 3 h post-prandial and may explain the reduced dietary endotoxemia we have previously reported [2]. Immune response in the gastrointestinal tract requires transmigration of various circulating leukocytes to the site of infection/damage, which is mediated by interleukin 7 (IL7); Caspase recruitment domain family, member 9 (CARD9); and Fc fragment of IgE, high affinity I, receptor for gamma polypeptide (FCER1G) [28,29,30,31]. IL7 mediates T-cells’ transmigration from circulation to the gastrointestinal tract [28], which typically occurs in response to dietary endotoxemia. CARD9 expression in gastrointestinal tract neutrophils regulates gut microbiota and resistance to gastrointestinal tract inflammation and colitis [29,30]. FCER1G expression regulates the density of group 3 innate lymphoid cells (ILC3) in the gastrointestinal tract to maintain mucosal barrier immunity and regulation of the gut inflammatory state [31]. Also, ILC3 concentration is substantially increased in the gastrointestinal tract of individuals with active chronic disease [32]. In the present study, probiotic supplementation increased the expression of IL7 (3 h), CARD9 (5 h), and FCER1G (3 h and 5 h); given their respective importance to gut barrier immunity, these appear to be a beneficial adaptation. It is interesting that we observed initial changes in IL7 and FCER1G at 3 h, followed by maintenance of FCER1G and increased CARD9 at 5 h. The post-prandial response observed in the present study may reflect an initial strengthening of the gut mucosal barrier, followed by the development of favorable gut microbiota by neutrophils. Collectively, these changes may reflect protection against post-prandial gastrointestinal inflammation and dietary endotoxemia. The transient timing of the observed responses warrants future studies given their potential implications for long-term gastrointestinal health.

### 4.3. Future Risk of IBD

We identified seven mRNAs (PD-L1, CSF1R, FAS, BID, FADD. GATA3, and KIR3DL) whose expression was significantly altered during the post-prandial period following probiotic supplementation and may be associated with future risk of IBD. Programmed Death Ligand 1 (PD-L1) positive neutrophils play a key role in combatting bacterial infection and resolving infection-associated inflammation [33], and PD-L1 is a common pharmaceutical treatment target to blunt secondary colitis [34]. Colony stimulating factor 1 receptor (CSF1R) works in tandem with PD-L1 to mediate monocytes’ maturation into resident intestinal macrophage [35]. Patients with active IBD and decreased CSF1R expression have an increased risk of developing colon cancer [36]. In the present study, we observed an increase in both PD-L1 (3 h and 5 h) and CSF1R (5 h) expression with probiotic use, which may represent novel findings. To our knowledge, no published studies have reported that probiotic supplementation increased post-prandial PD-L1 and CSF1R expression. Given the long-term importance of PD-L1 and CSF1R expression to the management of macrophages and inflammation, it seems reasonable to speculate that the observed changes may reflect prophylactic probiotic protection from future risk of IBD.

TNF receptor superfamily member 6 (FAS) is one of 15 senescence-associated secretory proteins (SASPs), and chronically elevated expression of FAS has been associated with frailty and reduced ability to respond to infectious challenge [37]. FAS has also been implicated in the progression of IBD and its effects are mediated by BH3-interacting domain death agonist (BID) [38]. Specifically, a loss of BID reduced neutrophil apoptosis in the gastrointestinal tract [39], increasing the risk of inflammatory disease, such as IBD [40]. In the present study, we initially observed a significant increase in FAS (3 h), followed by BID (5 h) expression. Given their respective and connected roles in regulating neutrophile apoptosis in the gastrointestinal tract, it is reasonable to speculate that the effect we observed post-prandial following probiotic supplementation is beneficial in nature. Specifically, the combined change in FAS and BID expression with probiotic use may translate to a reduction in future IBD risk due to improved gastrointestinal neutrophil turnover.

Fas (TNFRSF6)-associated via death domain (FADD) is a promoter of hypoxia-inducible factor-1 (HIF-1) expression, which is required to maintain gut barrier function and resist TNF-alpha-induced IBD onset [41]. In the present study, we observed increased FADD expression at 5 h with probiotic use. Chronic overexpression of GATA binding protein 3 (GATA3) can lead to the development of ulcerative colitis [42], thus making GATA3 a common pharmaceutical target for the treatment of IBD [43]. Chronic elevation of GATA3 is linked to allergies and a less ideal gastrointestinal immune response [44]; however, it is unclear what transient post-prandial elevations, such as those observed in the present study, truly mean. The present study showed similar increased GATA3 expression with probiotic supplementation at 5 h. While chronic changes in GATA3 expression relate to IBD progression, more research is needed to understand how transient changes, such as those observed in the present study, are involved. Different phenotypes of the killer cell immunoglobulin-like receptor, three domains, long cytoplasmic tail, 1 (KIR3DL1) gene are associated with susceptibility to IBD [45]; however, according to the published literature, it is unknown if transient changes in KIR3DL1 mRNA expression directly contribute to preventing IBD. In the present study, we observed a significant increase in KIR3DL1 expression at 5 h with probiotic use. We speculate that the combined changes in PD-L1, CSF1R, FAS, BID, FADD. GATA3, and KIR3DL expression following probiotic supplementation may reflect a potentially reduced risk of IBD onset and progression.

### 4.4. Study Limitations

While the present findings may be promising regarding the relationship between oral probiotic supplementation and gastrointestinal health, like any study, this study does have limitations that we acknowledge and will address in future studies. It is important to note that none of the participants measured in this study had IBD or any other diagnosed chronic disease. So, while we measured the change in mRNA expression, which may be associated with gastrointestinal health, none of our participants experienced a change in their health status. While we screened participants for dietary endotoxemia at baseline to create the sub-groups using our previously published criteria [2], we did not measure dietary endotoxemia after the probiotic intervention because the sample size would not have been sufficient for that outcome measure. Despite this, the intervention model is identical to the one we previously reported to reduce dietary endotoxemia, so we speculate that the effect would have been the same in this subset. The subset of participants included in the present study may seem small; however, the number tested was greater than our a priori sample size estimate (see methods above). We calculated the sample size based on mRNA expression, while guarding against false discovery rate (FDR). Also, the sample size we used in the present study is very similar to one that we have used in other studies where Nanostring mRNA measurements were a key outcome [1,15,16,17,18,19]. Any study that involves many dependent variables may result in observed ‘statistically significant’ effects that are simply chance, with a one-in-twenty likelihood of a ‘significant’ effect associated with a *p*-value of 0.05. The fact that so many of the observed changes were interrelated and have been previously attributed to gastrointestinal health leads us to conclude that our observed changes were real effects and not simply due to chance. We also include a robust set of methods to guard against identification of false positives while limiting false negatives. Future studies should seek to confirm our present observations using a larger sample size and possibly patients with compromised gastrointestinal health.

## 5. Conclusions

The key findings of the present study were that 45 d of spore-based probiotic supplementation was associated with a significant change in post-prandial expression of 15 mRNAs associated with gastrointestinal tract barrier function (four mRNAs: BATF3, CCR6, CXCR6, and PDCD2), gastrointestinal immunity (four mRNAs: CLEC5A, IL7, CARD9, and FCER1G), and future IBD risk (eight mRNAs: PD-L1, CSF1R, FAS, BID, FADD, GATA3, and KIR3DL). We speculate that the combined findings support the hypothesis that 45 d of probiotic supplementation was associated with an enhanced post-prandial immune response following eating a high-fat, high-calorie meal. Given the detrimental chronic effects of accumulated dietary endotoxemia, probiotic supplementation may represent an effective countermeasure. The findings contribute to previously published probiotic studies from our laboratory [1,2] and provide future directions for the continued study and identification of the effects associated with probiotic supplementation. Since we found the changes in healthy participants, the next logical step would be to determine if probiotic supplementation is effective in individuals with gastrointestinal tract dysfunction and/or disease.

## Data Availability

The raw data supporting the conclusions of this article will be made available by the authors on request.
